# Advisory Committee on Immunization Practices Recommended Immunization Schedule for Adults Aged 19 Years or Older — United States, 2019

**DOI:** 10.15585/mmwr.mm6805a5

**Published:** 2019-02-08

**Authors:** David K. Kim, Paul Hunter

**Affiliations:** ^1^Immunization Services Division, National Center for Immunization and Respiratory Diseases, CDC; ^2^University of Wisconsin School of Medicine and Public Health, Madison, Wisconsin.

In October 2018, the Advisory Committee on Immunization Practices (ACIP)* voted to recommend approval of the Recommended Immunization Schedule for Adults, Aged 19 Years or Older, United States, 2019. The 2019 adult immunization schedule, available at https://www.cdc.gov/vaccines/schedules,^†^ summarizes ACIP recommendations in two tables and accompanying notes. The 2019 adult immunization schedule has been approved by the CDC Director, the American College of Physicians, the American Academy of Family Physicians, the American College of Obstetricians and Gynecologists, and the American College of Nurse-Midwives.

ACIP’s recommendations on use of each vaccine are developed after in-depth reviews of vaccine-related data, including disease epidemiology and burden, vaccine efficacy and effectiveness, vaccine safety, quality of evidence, feasibility of program implementation, and economic analyses of immunization policy (*1*). The adult immunization schedule is published annually to consolidate and summarize updates to ACIP recommendations on vaccination of adults and assist health care providers in implementing current ACIP recommendations. The use of trade names of vaccines in this report and in the adult immunization schedule is for identification purposes only and does not imply endorsement by ACIP or CDC.

For further guidance on the use of vaccines in the adult immunization schedule, health care providers should refer to the full ACIP recommendations at https://www.cdc.gov/vaccines/hcp/acip-recs/index.html. Changes in recommended use of vaccines can occur between annual updates to the adult immunization schedule. These changes, if made, are available at https://www.cdc.gov/vaccines/acip/recommendations.html.^§^ Printable versions of the 2019 adult immunization schedule and instructions for ordering printed copies are available at https://www.cdc.gov/vaccines/schedules/hcp/adult.html#note.

The 2019 adult immunization schedule is a product of extensive formal usability testing of 2017 and 2018 adult immunization schedules, including in-depth interviews with 48 primary care physicians, nurse practitioners, physician assistants, pharmacists, nurses, and medical assistants, who reported being familiar with the adult immunization schedule, and an Internet survey of 251 internal medicine and family medicine physicians to assess their impressions and preferences on redesigned drafts of the adult immunization schedule (*2*). In addition to incorporating new ACIP recommendations on influenza, hepatitis A, and hepatitis B vaccinations, each vaccination section in the 2019 adult immunization schedule was revised for clarity, brevity, and, for vaccines that also appear in the 2019 child and adolescent immunization schedule (*3*), consistency between the two schedules. Because usability testing found that providers rarely used the table of contraindications and precautions for vaccines recommended for adults that was a part of previous iterations of the adult immunization schedule, the table was removed from the 2019 adult immunization schedule. Information on vaccine contraindications and precautions is available at https://www.cdc.gov/vaccines/hcp/acip-recs/general-recs.

## Changes in the 2019 Adult Immunization Schedule: Updated ACIP Recommendations

**Influenza Vaccination**. In June 2018, ACIP updated recommendations on the use of live attenuated influenza vaccine (LAIV) (FluMist Quadrivalent, AstraZeneca) after two influenza seasons (2016–17 and 2017–18), during which use of LAIV was not recommended in the United States (*4*). For the 2018–19 season, any licensed influenza vaccine that is recommended for age and health status of the patient may be used. LAIV is an option for adults aged ≤49 years, except those who 1) have immunocompromising conditions, including human immunodeficiency virus (HIV) infection; 2) have anatomic or functional asplenia; 3) are pregnant; 4) have close contact with or are caregivers of severely immunocompromised persons in a protected environment; 5) have received influenza antiviral medications in the previous 48 hours; or 6) have a cerebrospinal fluid leak or a cochlear implant. Adults with a history of Guillain-Barré syndrome within 6 weeks of receipt of a previous dose of influenza vaccine generally should not receive influenza vaccine.

**Hepatitis B Vaccination**. In February 2018, ACIP recommended use of the new single-antigen recombinant hepatitis B vaccine with a novel cytosine-phosphate-guanine 1018 oligodeoxynucleotide adjuvant (Heplisav-B, Dynavax) for prevention of hepatitis B virus infection in adults aged ≥18 years (*5*). Approved by the Food and Drug Administration in November 2017, Heplisav-B is routinely administered in 2 doses given ≥4 weeks apart. It can be used as a substitute in a 3-dose series with a different hepatitis B vaccine, but a valid 2-dose series requires 2 doses of Heplisav-B with ≥4 weeks between doses. When feasible, a vaccine from the same manufacturer should be used to complete the vaccination series. However, vaccination should not be deferred if the previously administered hepatitis B vaccine is unknown or if a vaccine from the same manufacturer is not available. A pregnant woman with an indication for hepatitis B vaccination should not receive Heplisav-B because no safety data are available on its use during pregnancy.

**Hepatitis A Vaccination**. In October 2018, ACIP recommended adding homelessness as an indication for routine hepatitis A vaccination with a 2-dose series of single-antigen hepatitis A vaccine (Havrix, GlaxoSmithKline; Vaqta, Merck) or a 3-dose series of combination hepatitis A and hepatitis B vaccine (Twinrix, GlaxoSmithKline) (*6*). Other populations at increased risk for hepatitis A virus infection or severe hepatitis A disease and recommended to receive vaccination include 1) persons with chronic liver disease or clotting factor disorders; 2) travelers in countries with high or intermediate hepatitis A endemicity; 3) persons with close personal contact with an international adoptee in the first 60 days after arrival from a country with high or endemic hepatitis A prevalence; 4) men who have sex with men; 5) persons who use injection or noninjection drugs; and 6) persons who work with hepatitis A virus in a laboratory or with nonhuman primates infected with the virus (*7*–*9*). In addition, any person who is not at risk for hepatitis A virus infection but wants protection against it may be vaccinated.

## Changes in the 2019 Adult Immunization Schedule: Revised Content, Format, and Graphics

**Cover. Recommended Adult Immunization Schedule**. The cover page of the 2019 adult immunization schedule has been simplified and contains the following changes:

- Features a shortened title, provides basic instructions on how to use the adult immunization schedule to systematically identify vaccination needs of adults, and lists routinely recommended vaccines and their standardized abbreviations and trade names.
- Includes web links through which health care providers can download the CDC Vaccine Schedules App and access reference materials on surveillance of vaccine-preventable diseases, including case identification and disease outbreak response.
- Simplifies instructions for reporting suspected cases of reportable vaccine-preventable diseases to local or state health departments and for reporting postvaccination adverse events and serious adverse events to the Vaccine Adverse Event Reporting System; information on the Vaccine Injury Compensation Program; and links to other resources, such as Vaccine Information Statements and recommended vaccines for travelers.

**Table 1. Recommended Adult Immunization Schedule by Age Group**. Table 1 (previously known as Figure 1) describes routine and catch-up vaccination recommendations for adults by age. ACIP recommends routine annual influenza vaccination for all persons aged ≥6 months who do not have contraindications; 1 annual dose of IIV, RIV, or LAIV that is appropriate for age and health status of the vaccine recipient is recommended. Table 1 contains the following change:

- Lists LAIV separately from inactivated influenza vaccine (IIV) (many branded products) and recombinant influenza vaccine (RIV) (Flublok Quadrivalent, Sanofi Pasteur) for adults aged ≤49 years.

**Table 2. Recommended Adult Immunization Schedule by Medical Condition and Other Indications.** Table 2 (previously known as Figure 2) describes indications for which vaccines, if not previously administered, should be administered unless noted otherwise. Table 2 contains the following changes:

- Lists LAIV separately from IIV and RIV.
- Contains two new display colors for some vaccines: orange and pink. Orange indicates “Precaution—vaccine might be indicated if benefit of protection outweighs risk of adverse reaction”; pink indicates “Delay vaccination until after pregnancy if vaccine is indicated.”
- Designates the use of LAIV in pregnant women and immunocompromised adults, including those with HIV infection, as “Contraindicated—vaccine should not be administered because of risk for serious adverse reaction” (red). The risk for associated adverse effects from the use of LAIV in adults with functional or anatomic asplenia or complement deficiencies is not known; however, the use of LAIV in this population has also been designated as “Contraindicated” (red). For adults with end-stage renal disease, heart or lung disease, chronic liver disease, or diabetes, the use of LAIV has been given the “Precaution” (orange) designation.
- Designates the use of serogroup B meningococcal vaccine (MenB) (Bexsero, GlaxoSmithKline; Trumenba, Pfizer) in pregnant women as “Precaution” (orange). MenB should be deferred in pregnant women unless they are at increased risk for serogroup B meningococcal disease and the benefits of vaccination are considered to outweigh potential risks (*10*).
- Maintains the use of meningococcal serogroups A, C, W-135, and Y conjugate vaccine (MenACWY) (Menactra, Sanofi Pasteur; Menveo, GlaxoSmithKline) in pregnant women as “Recommended vaccination for adults with an additional risk factor or another indication” (purple). In contrast to the recommendation to defer administration of MenB vaccine to pregnant women, pregnancy should not preclude the use of MenACWY vaccine if it is otherwise indicated (*11*).
- Designates the use of human papillomavirus (HPV) vaccine (Gardasil 9, Merck) and recombinant zoster vaccine (RZV) (Shingrix, GlaxoSmithKline) in pregnant women as “Delay until after pregnancy” (pink). The use of HPV vaccine is not recommended for pregnant women (*12*,*13*). Pregnant women should consider delaying receipt of RZV, if it is indicated, until after pregnancy (*14*). Live attenuated zoster vaccine (Zostavax, Merck) is contraindicated during pregnancy (*15*).

**Notes. Recommended Adult Immunization Schedule.** Each routinely recommended vaccine for adults in Tables 1 and 2 is accompanied by notes (previously known as footnotes), designed to provide additional information about routine vaccination and recommendations in special situations. The notes contain the following format changes:

- Lists vaccination sections alphabetically (superscript footnote numbers in the former figures [now tables] have been removed).
- Contains concise information describing vaccine indications, dosing frequencies and intervals, and other published ACIP recommendations for each section.
- Includes new recommendations on influenza, hepatitis B, and hepatitis A vaccines in their respective sections.
- Removes recommendations for vaccination in outbreak settings in measles, mumps, and rubella, and meningococcal vaccination notes.

## Additional Information

The Recommended Adult Immunization Schedule, United States, 2019, is available at https://www.cdc.gov/vaccines/schedules/hcp/adult.html and in the Annals of Internal Medicine (*16*). The full ACIP recommendations for each vaccine are also available at https://www.cdc.gov/vaccines/hcp/acip-recs/index.html. All vaccines identified in Tables 1 and 2 (except zoster vaccines) also appear in the Recommended Immunization Schedule for Children and Adolescents, United States, 2019 (*3*). The notes for vaccines that appear in both the adult immunization schedule and the child and adolescent immunization schedule have been harmonized to the greatest extent possible.

## Footnotes

* Recommendations for routine use of vaccines in children, adolescents, and adults are developed by ACIP. ACIP is chartered as a federal advisory committee to provide expert external advice and guidance to the CDC Director on use of vaccines and related agents for the control of vaccine-preventable diseases in the civilian population of the United States. Recommendations for routine use of vaccines in adults are approved by the American Academy of Family Physicians, American College of Nurse-Midwives, American College of Obstetricians and Gynecologists, and American College of Physicians. ACIP recommendations approved by the CDC Director become agency guidelines on the date published in the *Morbidity and Mortality Weekly Report*. Additional information about ACIP is available at https://www.cdc.gov/vaccines/acip.

† Past immunization schedules are available at https://www.cdc.gov/vaccines/schedules/past.html.

§ CDC encourages organizations that previously have relied on copying the adult immunization schedule on their websites to use syndication instead, as a more reliable method for displaying the most current and accurate adult immunization schedule. Use of content syndication requires a one-time step that ensures an organization's website displays the adult immunization schedule as soon as it is published or revised. The syndication code for the adult immunization schedule and instructions for its use can be found at https://www.cdc.gov/vaccines/schedules/syndicate.html. CDC also offers technical assistance for implementing this form of content syndication (e-mail request to ncirdwebteam@cdc.gov). Changes in ACIP recommendations in the adult immunization schedule before the next scheduled annual update, if any, are available at https://www.cdc.gov/vaccines/schedules/hcp/adult.html.
